# Dynamic Kinematic Assessment with 3D Motion Analysis After Arthroscopic Bankart Repair: A Mid- to Long-Term Study

**DOI:** 10.3390/jcm14228204

**Published:** 2025-11-19

**Authors:** Pit Hetto, Raissa Liewald, David M. Spranz, Stefanos Tsitlakidis

**Affiliations:** 1 Department of Orthopaedic Surgery, University of Heidelberg, Schlierbacher Landstraße 200a, 69118 Heidelberg, Germany; 2 Department for Pneumology and Critical Care Medicine, Thoraxklinik Heidelberg, Röntgenstraße 1, 69126 Heidelberg, Germany

**Keywords:** arthroscopic Bankart repair, shoulder instability, three-dimensional motion analysis, range of motion, activities of daily living

## Abstract

**Background/Objectives:** The aim of the study is to first evaluate mid- to long-term changes in shoulder range of motion (ROM) and functional performance in activities of daily living (ADLs) after arthroscopic Bankart repair using three-dimensional (3D) motion analysis. **Methods:** We prospectively analyzed five patients (mean age: 31.8 years) pre- and postoperatively at 8.4 months and eight patients retrospectively at 12.1 years (mean age: 40.4 years). Fifteen asymptomatic adults served as controls. Shoulder kinematics were assessed using the Heidelberg Upper Extremity (HUX) model during maximum ROM and four ADL tasks (apron, neck, wash, and book). **Results:** At short-term follow-up, forward flexion improved by 31° (*p* < 0.05) and abduction improved by 70° (*p* < 0.05), while other movements showed non-significant trends toward improvement. Long-term follow-up demonstrated sustained or increased gains in flexion (+9°) and abduction (+7°) but significant declines in external rotation (−5°) and internal rotation (−30°). ADL analyses showed significant postoperative gains in abduction/adduction during “apron” (+6.7°) and “neck” (+49.8°) tasks. The long-term results remained comparable to or better than postoperative values in most planes, although external/internal rotation during the “wash” task decreased over time. Compared with normative controls, patients employed a larger ROM during some ADLs, suggesting compensatory mechanisms. **Conclusions:** Arthroscopic Bankart repair yields sustained mid- to long-term improvements in shoulder ROM and ADL performance. Rotational deficits persist despite maintained flexion and abduction in the long run, underscoring the need for targeted rehabilitation strategies.

## 1. Introduction

The accurate and reliable measurement of shoulder range of motion (ROM) is essential for clinical assessment and postoperative evaluation following therapeutic interventions in shoulder pathology [1,2]. In routine orthopedic practice, manual goniometry remains the gold standard for joint angle assessment, including the shoulder. This method is simple, widely accessible, and cost-effective, and it enables the evaluation of both active and passive motion. However, its accuracy is limited to ±5–10° and is highly examiner-dependent [3,4,5]. Moreover, goniometric measurements offer only static, two-dimensional snapshots of joint angles and are insufficient for isolating glenohumeral motion from compensatory movements involving the thorax, spine, and shoulder girdle, thus representing an inability to capture complex joint movements [5,6,7,8].

Three-dimensional (3D) marker-based motion analysis has emerged as a promising tool for capturing both static and dynamic shoulder motion. Already well established in lower-extremity gait analysis [9,10], this non-invasive technique offers examiner-independent, reproducible measurements of joint kinematics at any time point throughout the motion cycle [11]. However, the application of this technology to the upper extremity presents unique challenges due to the inherent complexity of shoulder motion [12]. Lower extremity movements are typically repetitive and occur within defined planes, while shoulder motion involves multiple degrees of freedom across three spatial planes, lacks periodicity, and exhibits high interindividual variability [13,14]. These factors limit reproducibility and increase susceptibility to soft-tissue artifacts caused by marker occlusion, particularly during large arc movements.

To address these limitations, marker clusters—composed of three or more markers placed on rigid anatomical landmarks—are used to create technical coordinate systems. When combined with defined anatomical reference points, these clusters allow for post hoc reconstruction of joint kinematics and minimize soft tissue artifacts [15,16]. The “Heidelberg Upper Extremity (HUX) Model,” developed by the Upper Extremity Motion Analysis Group at the Heidelberg University Orthopedic Department, enables the precise calculation of the shoulder joint center at any point during motion [17]. This model allows for the functional determination of dynamic joint axes and the differentiation of glenohumeral from scapulothoracic motion. Its validity and reproducibility have been thoroughly evaluated [17,18,19].

By enabling accurate assessments of complex 3D movement patterns, this method facilitates the use of motion analysis as a valuable quality control tool in shoulder rehabilitation [20].

The glenohumeral joint is the most mobile joint in the human body due to the small articular contact surface—only 25–30% congruence between the humeral head and glenoid—and the capacious joint capsule [21]. Joint stability is maintained by a dynamic balance between passive (capsuloligamentous) and active (musculature of the trunk and lower and upper extremities) stabilizers [21,22,23]. Shoulder instability is defined as the abnormal translation of the humeral head relative to the glenoid during active movement, reflecting a failure to maintain centered articulation [24].

The incidence of anterior shoulder dislocation is 23 per 100,000 person-years, with anterior–inferior instability accounting for 80% of cases, followed by posterior and multidirectional instability at 10% each [25]. In young patients, traumatic anterior dislocation is most common, typically involving injury to the inferior glenohumeral ligament complex [26]. Male patients are more frequently affected, although the proportion of female patients increases between ages 35 and 40 [25,26].

Arthroscopic Bankart repair—refixation of the capsulolabral complex—is currently the standard treatment for first-time traumatic anterior dislocation in young, active individuals [27,28,29,30]. Additional indications include chronic, post-traumatic recurrent instability with or without hyperlaxity [29]. Despite advancements in surgical technique and anchor systems, redislocation rates remain between 15% and 25% postoperatively [31,32]. Identified risk factors for recurrence include young age (<20 years), glenoid bone loss, Hill–Sachs lesions, and generalized hyperlaxity [32].

The aim of this study was to assess postoperative and mid-term changes in active maximum range of motion (ROM) and their functional translation into activities of daily living (ADLs) following Bankart repair in patients with shoulder instability using three-dimensional motion analysis. We hypothesized that improvements in both ADL performance and ROM during ADLs would be sustained over time, with only minimal decline at mid-term follow-up.

## 2. Materials and Methods

### 2.1. Study Design and Ethics

We conducted a two-arm study including a prospective short-term and a retrospective long-term cohort of patients who underwent arthroscopic Bankart repair. A normative control group was also analyzed. All participants provided informed consent prior to testing. This study was approved by the local Ethics Committee (reference S-456/2018) in accordance with the Declaration of Helsinki of 1975, as revised in 2013. This study was conducted as an exploratory study to determine whether the previous results of the HUX model were transferable to patients with shoulder instability.

### 2.2. Prospective Cohort

The prospective arm included five male patients (mean age: 31.8 years; SD: 11.2) treated with arthroscopic Bankart repair for chronic anterior shoulder instability. Three-dimensional (3D) motion analysis was performed preoperatively and repeated at a mean of 8.4 months (SD 3.5) after surgery as part of clinical follow-up. Four of five patients were right-handed, and in four cases, the non-dominant shoulder was affected.

### 2.3. Long-Term Cohort

The retrospective arm included eight patients (five male and three female) with a mean age of 40.4 years (SD 10.3) at the time of surgery. All underwent primary arthroscopic Bankart repair, with revision procedures excluded. The mean follow-up period was 12.1 years (SD 1.6). Seven of eight patients were right-handed, and in seven cases, the dominant shoulder was affected.

### 2.4. Control Group

A normative control group consisted of 15 asymptomatic adults (12 male and 3 female; mean age: 33.7 years; SD: 12.2). Participants had no history of upper extremity injury, musculoskeletal impairment, or spinal/neck pathology. The exclusion criteria included any current pain or muscular tension and participation in competitive sports. In some patients, the contralateral, asymptomatic shoulder was also used as an internal reference.

### 2.5. Motion Analysis Protocol

Kinematic data were acquired in the motion laboratory of the Department of Orthopaedic Surgery, Heidelberg University, using a Vicon motion capture system (Vicon Motion Systems Ltd., Oxford, UK). The setup included 12 infrared cameras operating at 120 Hz, with a measurement accuracy of ±1 mm and a resolution of 4 megapixels per camera. Prior to each session, the system was calibrated according to manufacturer guidelines.

Reflective markers (8 mm diameter) were placed according to the validated Heidelberg Upper Extremity (HUX) model, which allows the functional calculation of the glenohumeral joint center and separation of glenohumeral from scapulothoracic motion, in accordance with the guidelines of the International Society of Biomechanics [17,19,33,34,35,36]. Single spherical markers were placed over defined bony landmarks (spinous processes of C7 and T8, jugular notch, sternum, xiphoid process, scapular spine, and dorsal forearm). In addition, four cluster marker sets (two reflective spheres mounted on a 40 mm rod) were applied bilaterally to the acromion, humeral deltoid tuberosity, olecranon, and distal dorsal forearm. This configuration allowed reliable tracking while minimizing soft tissue artefact.

### 2.6. Measurement Protocol

#### 2.6.1. Calibration and Baseline

Data collection began with calibration recordings in a seated position with arms resting at the sides and hands on the knees.

#### 2.6.2. Maximum Range of Motion (ROM)

Participants then performed active maximal shoulder movements from neutral positions: flexion, extension, abduction, internal rotation (IRO), and external rotation (ARO) (arm at side), as well as elbow flexion/extension and forearm pronation/supination. Each trial was repeated three times.

#### 2.6.3. Activities of Daily Living (ADLs)

To assess functional mobility, four standardized ADL tasks were performed five times per side:

1. Apron tying (apron)—reaching posteriorly with the hand to place the palm centrally on the back.
2. Neck grip (neck)—reaching to the nape of the neck at the hairline.
3. Opposite axilla wash (wash)—simulating washing the contralateral axilla with a cloth.
4. Book reaching (book)—reaching at eye level to grasp a book on an adjustable shelf.

#### 2.6.4. Activity-Related Range of Motion (AROM)

For each ADL, the angular range between the highest and lowest recorded values during the movement cycle was calculated, providing a task-specific measure of dynamic shoulder mobility.

#### 2.6.5. Statistical Analysis

Kinematic data were exported and processed using MATLAB (Version R2013a; The MathWorks Inc., Natick, MA, USA). Statistical analyses were performed with Microsoft Excel (Office 2019, Version 2305; Microsoft Corporation, Redmond, WA, USA) and SPSS Statistics (Version 20; SPSS GmbH Software, Munich, Germany). Data distribution was assessed using the Shapiro–Wilk test. Levene’s test was applied to evaluate the homogeneity of variance. Differences in ROM in the prospective group were analyzed using a dependent samples *t*-test. Between-group differences in ROM were analyzed using independent samples *t*-tests. The minimal clinically important difference (MCID) was estimated using a distribution-based method based on the standard deviation [37,38]. A *p*-value of <0.05 was considered statistically significant.

## 3. Results

### 3.1. Short-Term Postoperative Outcomes

Comparison of preoperative and postoperative data (mean follow-up of 8.4 months) demonstrated no significant improvements in the shoulder range of motion (ROM) (Table 1):

- Forward flexion increased by 58° (from 90° to 148°).
- Abduction improved by 70° (from 76° to 146°).
- Other movements (extension, external and internal rotation, elbow motion, and pronation/supination) showed non-significant trends toward improvement.

External rotation increased by 16° (from 3° to 19°) and internal rotation by 16° (from 39° to 55°) but did not reach statistical significance.

The MCID was calculated for each movement and is listed in Table 1.

Clinically meaningful improvements were observed in shoulder flexion and abduction, as both exceeded their respective MCID thresholds. Elbow flexion/extension and internal rotation showed changes slightly above the MCID, suggesting potential clinical relevance, though less pronounced. In contrast, shoulder extension, external rotation, and forearm pro-/supination did not reach the MCID threshold and are therefore considered not clinically relevant.

These findings confirm substantial short-term gains, particularly in flexion and abduction, with generalized improvement in most other planes.

### 3.2. Long-Term Outcomes

**Figure 1:**
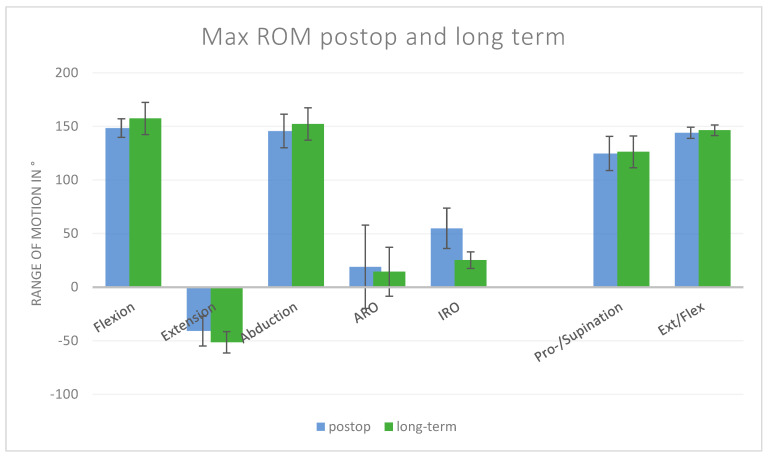
**Comparison of postoperative and long-term maximum values**. Flexion, extension, abduction, ARO, and IRO are shoulder movements; pro-/supination and ext/flex are movements of the elbow.

At a mean of 12.1 years post-surgery, the range of motion remained improved compared with the preoperative state, with some further gains compared to early postoperative values (Table 2 and Figure 1).

- Forward flexion improved by an additional 9° compared to postoperative values (148° vs. 157°).
- Abduction increased by 7° (146° vs. 152°).
- Extension tended to increase by 10° (non-significant).

However, rotational movements deteriorated:

- External rotation decreased by 5° (19° vs. 14°).
- Internal rotation decreased by 30° (55° vs. 25°; *p* < 0.05).

Thus, while flexion and abduction were sustained or improved, long-term deterioration was evident in rotational planes.

#### Comparison of Preoperative, Postoperative, and Long-Term Individual Ranges of Motion in Daily Activities After Bankart Repair to Normative Values

**Figure 2:**
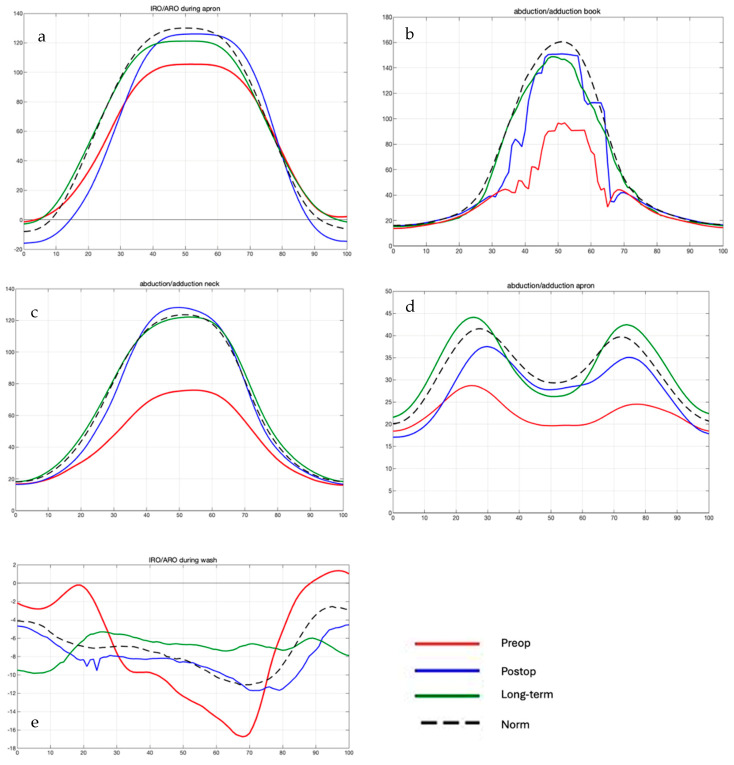
Motion over time with angle in ° over % of movement. (**a**) represents rotational movement during the apron task, (**b**) abduction/adduction during the book task, (**c**) abduction/adduction during the neck task, (**d**) abduction/adduction during the apron task, and (**e**) IRO/ARO during the wash task. The red line is the preoperative group, the blue line is the postoperative group, the green line is the long-term group, and the black line is the normative group. The pre- to postoperative improvements in all motions, except the IRO/ARO during wash, can be seen. No significant difference between the postop, long-term, and norm groups.

Overall, Bankart repair tended to improve all individual ROMs in the pre- and postoperative patient cohort, except for external/internal rotation during the wash task (Figure 2). Significant improvements were observed in the abduction/adduction ROM for the daily apron and neck activities, with increases of 6.7° and 49.8°, respectively.

Comparisons of postoperative and long-term daily activity ROM revealed a significant difference in external/internal rotation during the wash task over time (16° decrease). Non-significant but trend-level differences were observed in external/internal rotation for the apron task (17.6°). Other ROMs tended to improve in the long-term follow-up.

**Figure 3:**
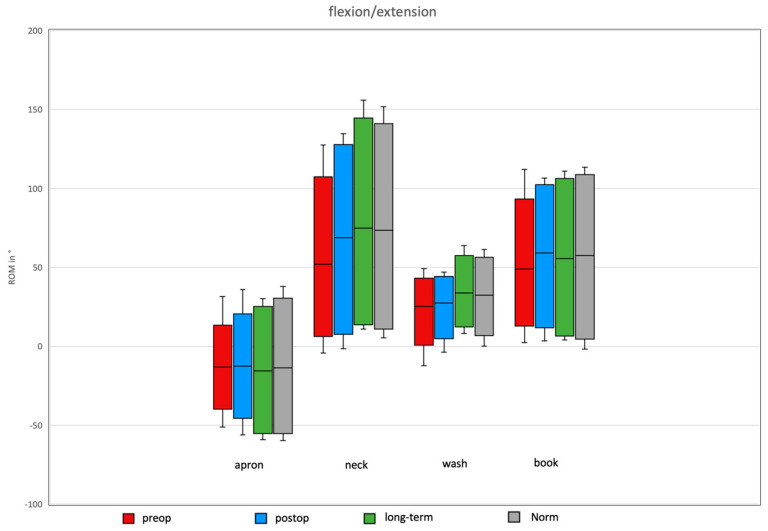
Flexion/extension of the shoulder for the four ADLs in the different groups. An increase in the ROM was observed in all movements for the prospective group, with no significant difference between the long-term and norm groups.

**Figure 4:**
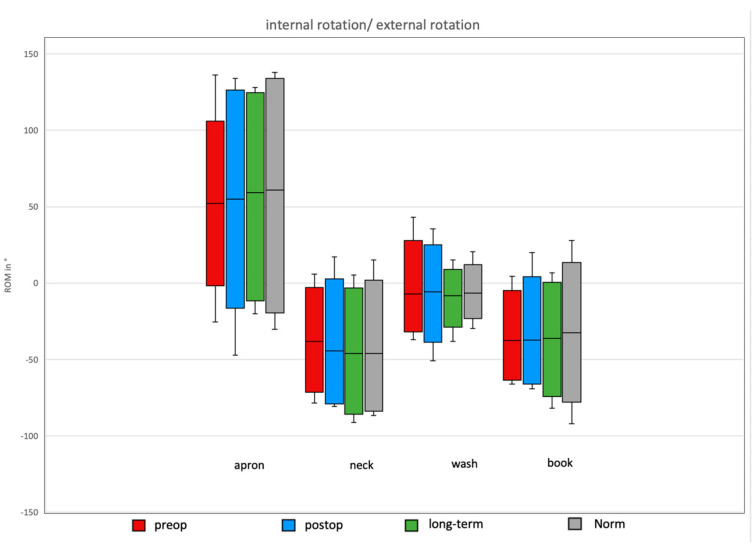
Rotational movement for the four ADLs in the different groups. A significant difference between the long-term and postop groups was observed for the wash task, as well as a non-significant decline during the apron task.

**Figure 5:**
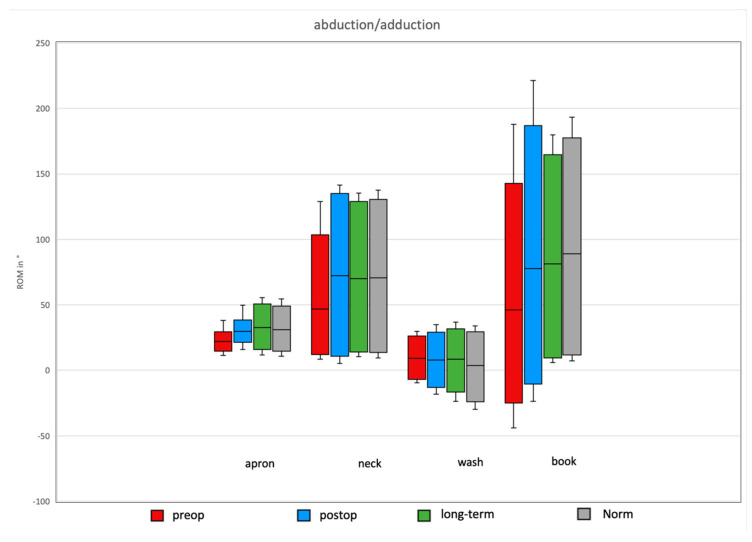
Adduction and abduction for the four ADLs in the different groups. Significant improvements for the daily apron and neck activities in the prospective group.

Further comparisons of postoperative daily activity ROM with normative values showed a significant increase in external/internal rotation for the wash task (17.7° higher than norms). Non-significant trend-level postoperative increases were observed in abduction/adduction for the book task (3.5°) and in external/internal rotation for the apron task (4°). For the remaining movements, ROM in the normative cohort tended to exceed that of the postoperative subjects (Figure 3, Figure 4 and Figure 5).

### 3.3. Daily Activity AROM in Ante-/Retroversion of the Shoulder After Bankart Repair

**Figure 6:**
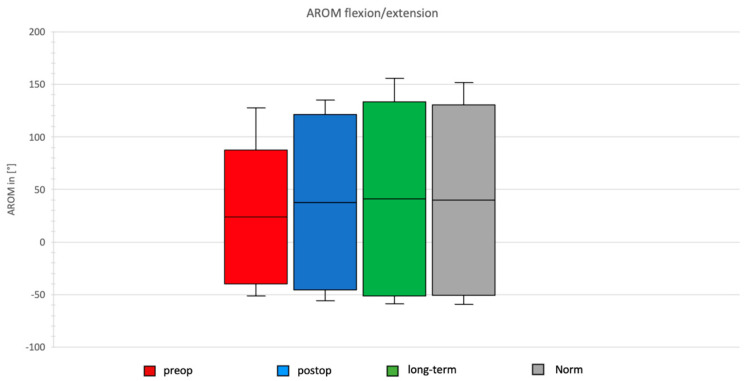
AROM for flexion and extension in the four groups.

In the preoperative group, AROM for ante-/retroversion was 127°, with maximal anteversion observed during the neck task and maximal retroversion during the apron task. Postoperatively, after Bankart repair, the total AROM in the ante-/retroversion plane increased to 166.59°, with maximal anteversion still during the neck task and maximal retroversion during the apron task (Figure 6).

The long-term group after Bankart repair demonstrated a total AROM of 185.1°, with maximal values again for anteversion during the neck task and retroversion during the apron task.

In the normative cohort, total AROM was 181.45°, with maximal anteversion during the neck task and maximal retroversion during the apron task.

### 3.4. Daily Activity AROM in Abduction/Adduction of the Shoulder

**Figure 7:**
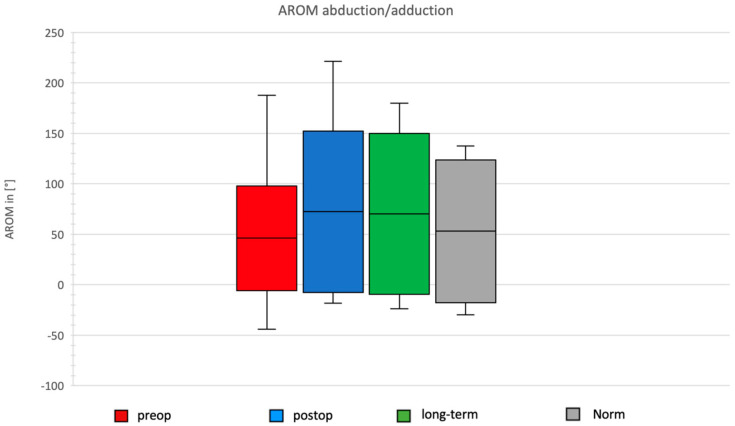
AROM for abduction and adduction in the four groups.

In the preoperative group, AROM for abduction/adduction was 103.76°, with the maximal range observed during the book task. Postoperatively, after Bankart repair, the total AROM in the abduction/adduction plane increased to 159.79°, with maximal abduction during the book task and maximal adduction during the wash task (Figure 7).

The long-term group after Bankart repair showed a total ROM of 159.31°, with maximal abduction during the book task and maximal adduction during the wash task.

In the normative cohort, the total ROM was 141.56°, with maximal abduction during the neck task and maximal adduction during the wash task.

### 3.5. Daily Activity AROM in External/Internal Rotation of the Shoulder

**Figure 8:**
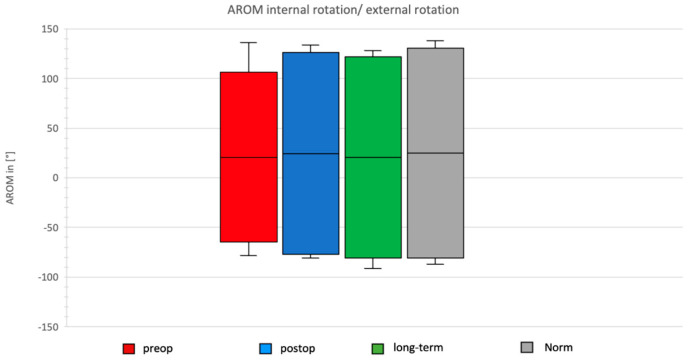
AROM for IRO and ARO in the four groups.

In the preoperative group, the AROM for external/internal rotation was 170.81°, with maximal external rotation during the neck task and maximal internal rotation during the apron task. Postoperatively, after Bankart repair, the total AROM in the external/internal rotation plane increased to 203.53°, with maximal external rotation during the neck task and maximal internal rotation during the apron task (Figure 8).

The long-term group after Bankart repair showed a total ROM of 202.33°, with maximal values again during neck and apron tasks.

In the normative cohort, the total ROM was 141.56°, with maximal abduction during the neck task and maximal adduction during the wash task.

## 4. Discussion

This study demonstrates that arthroscopic Bankart repair leads to substantial short-term improvements in the shoulder’s range of motion, particularly in forward flexion and abduction, as well as internal rotation and elbow flexion/extension. When compared with the published literature, the postoperative values in this study fall within the mid-range of reported outcomes for flexion and abduction, with forward flexion reported between 135° and 158° and abduction between 137° and 179° [39,40]. External rotation, however, was markedly lower than most reference values, despite a functional gain from the baseline. Previous studies have shown postoperative external rotation ranging from 45° to 61° [40,41,42], while the present study observed only 19° at short-term follow-up and 14° at long-term review. Nonetheless, this represents a meaningful improvement from preoperative values. A notable strength of this study is the use of three-dimensional motion analysis rather than goniometry. Conventional goniometric assessment is examiner-dependent and limited to static two-dimensional measurements [3,4,5], whereas 3D optical tracking with the validated HUX model allows examiner-independent, reproducible, and dynamic assessments of shoulder kinematics [17,18,19]. This methodological advantage may also explain discrepancies with previously reported values, particularly for rotational movements where definitions and measurement techniques differ substantially between studies.

Functional analysis during activities of daily living revealed that postoperative patients not only improved compared to their preoperative status but also frequently employed larger arcs of motion than healthy controls. Interestingly, external/internal rotation during the “wash” and “apron” tasks exceeded normative values postoperatively, while abduction during the “book” task showed a trend towards improvement compared to controls. This suggests that compensatory strategies may play a role in maintaining functional ability despite residual deficits. Such mechanisms may also help explain why many patients report satisfactory functional outcomes despite measurable limitations in specific planes of motion [43,44,45].

These gains remained stable or improved further at long-term follow-up, confirming the durability of surgical restoration in these planes. However, internal and external rotation deteriorated over time, which is in line with several reports highlighting persistent limitations in rotational function after Bankart repair [42,46,47,48]. Although the exact mechanisms for this decline remain unclear, capsuloligamentous scarring, altered neuromuscular control, and compensatory movement strategies may contribute to this phenomenon [49]. A possible explanation discussed in the literature for these significant deviations from the intended target values is that, following unilateral shoulder injuries or disorders, bilateral impairments of proprioception may occur—even when the contralateral, non-affected side shows no clinical limitations or abnormalities—resulting in subconscious movement restrictions [49]. The underlying mechanisms have not yet been fully investigated. It is possible that unilateral damage to mechanoreceptors affects the central neuromuscular control mechanisms [50]. Furthermore, the deterioration of rotational movements may partly be attributed to degenerative changes in the rotator cuff, which plays a crucial role in this plane of motion. The age-related degeneration of the rotator cuff is a well-documented phenomenon [51].

These changes, particularly the decline in rotational movements, suggest that special attention should be given to targeted exercises addressing these motions. Rotational movements should be continuously trained throughout the long-term rehabilitation period to prevent further deterioration.

A meta-analysis demonstrated that most patients who were previously physically active do not return to sports after shoulder injury or surgery [45]. The primary reason cited is fear of re-injury. The exact cause of this phenomenon remains unclear, but functional cerebral alterations are assumed to play a role [43,44,45]. Therefore, postoperative rehabilitation should not be limited to purely musculoskeletal therapy but should also incorporate interventions that influence cortical activation [43].

### Limitations

The main limitations of this study include its small sample size, which reduces statistical power, and the relatively short postoperative follow-up of the prospective cohort. The use of the contralateral shoulder and normative controls as reference standards may also introduce bias, particularly given that the normative values in this study deviated from published reference data. Nonetheless, the use of validated 3D motion analysis provides robust and detailed insights into dynamic shoulder kinematics, which are not captured by traditional clinical tools.

## 5. Conclusions

Arthroscopic Bankart repair results in significant short-term improvements in shoulder motion, particularly in forward flexion and abduction, which are maintained or further enhanced at long-term follow-up. While most planes of motion remained stable, internal and external rotation demonstrated a decline over time, suggesting that rotational deficits may persist or worsen despite overall functional recovery. Analyses of activities of daily living revealed that patients frequently employed greater ranges of motion than healthy controls, indicating compensatory mechanisms that may support function despite residual limitations. These findings highlight the value of three-dimensional motion analysis in capturing dynamic shoulder kinematics and underline the importance of rehabilitation strategies specifically targeting rotational capacity to optimize long-term outcomes after Bankart repair. Further research is warranted following this exploratory study, ideally involving larger patient cohorts, which may ultimately support the implementation of 3D motion analysis as a routine tool for postoperative follow-up.

## Figures and Tables

**Table 1:** Comparison of maximum range of motion [°] between preoperative and postoperative groups, including the mean difference with 95% confidence interval (CI). For shoulder movement, extension and flexion are two separated movements; elbow pro- and supination, as well as extension and flexion, are combined movements. MCID was calculated for each movement.

Movement	Preop	SD-Preop	Postop	SD-Postop	Mean Difference, 95% CI	SD Difference	*p*-Values	MCID
Flexion Shoulder	89.99	±62.37	148.46	±8.71	58.47 [−19.7; 136.7]	±62.98	0.10	31.2
Extension Shoulder	−38.49	±16.88	−41.07	±13.88	2.58 [−24.5; 29.7]	±21.85	0.81	8.4
Abduction	76.12	±61.47	145.66	±15.63	69.53 [−9.3; 148.3]	±63.43	0.07	30.7
ARO	2.54	±41.58	18.98	±38.87	16.44 [−54.3; 87.2]	±56.92	0.55	20.8
IRO	38.75	±23.34	54.86	±18.85	16.11 [−21.2; 53.4]	±30.00	0.29	11.7
Pro-/Supination	117.13	±17.74	124.67	±15.94	7.55 [−22.1; 37.1]	±23.85	0.51	8.9
Ext/Flex Elbow	125.13	±31.44	144.07	±5.18	18.93 [−20.7; 58.5]	±31.86	0.26	15.7

**Table 2:** Comparison of maximum range of motion between postoperative and long-term groups [°]. For shoulder movement, extension and flexion are two separate movements; elbow pro- and supination, as well as extension and flexion, are combined movements. Statistically significant difference (*p* < 0.05).

Movement	Post-Op	SD-Post-Op	Long-Term	SD-Long-Term	Mean Difference, 95% CI	SD Difference	*p*-Values
Flexion Shoulder	148.46	±8.71	157.40	±14.95	8.94 [–5.7, 23.6]	±17.30	0.20
Extension Shoulder	−41.07	±13.88	−51.45	±10.00	–10.38 [–27.9, 7.1]	±17.11	0.19
Abduction	145.66	±15.63	152.28	±15.16	6.62 [–13.7, 26.9]	±21.78	0.47
ARO	18.98	±38.87	14.37	±22.87	–4.61 [–53.9, 44.7]	±45.10	0.82
IRO	54.86	±18.85	25.21	±7.80	–29.65 [–54.3, –5.0]	±20.40	**<0.05**
Pro-/Supination	124.67	±15.94	126.24	±14.81	1.57 [–18.8, 22.0]	±21.76	0.86
Ext/Flex elbow	144.07	±5.18	146.38	±5.01	2.31 [–4.4, 9.0]	±7.20	0.45

## Data Availability

The datasets used and/or analyzed during the current study are available from the corresponding author upon reasonable request.
